# Integrative transcriptomic analysis in human and mouse model of anaphylaxis identifies gene signatures associated with cell movement, migration and neuroinflammatory signalling

**DOI:** 10.3389/fimmu.2022.1016165

**Published:** 2022-12-08

**Authors:** Matija Rijavec, Aleš Maver, Paul J. Turner, Keli Hočevar, Mitja Košnik, Amnah Yamani, Simon P. Hogan, Adnan Custovic, Borut Peterlin, Peter Korošec

**Affiliations:** ^1^ University Clinic of Respiratory and Allergic Diseases Golnik, Golnik, Slovenia; ^2^ Biotechnical Faculty, University of Ljubljana, Ljubljana, Slovenia; ^3^ Clinical Institute of Medical Genetics, University Medical Centre, Ljubljana, Slovenia; ^4^ National Heart and Lung Institute, Imperial College London, London, United Kingdom; ^5^ Medical Faculty, University of Ljubljana, Ljubljana, Slovenia; ^6^ Department of Pathology, Michigan Medicine, University of Michigan, Ann Arbor, MI, United States; ^7^ Mary H. Weiser Food Allergy Center (MHWFAC), Department of Pathology, Michigan Medicine, University of Michigan, Ann Arbor, MI, United States; ^8^ Faculty of Pharmacy, University of Ljubljana, Ljubljana, Slovenia

**Keywords:** transcriptome analysis, anaphylaxis, basophils, neutrophils, cell movement, migration, neuroinflammatory signaling, lipid activating nuclear receptors signaling

## Abstract

**Background:**

Anaphylaxis is an acute life-threatening allergic reaction and a concern at a global level; therefore, further progress in understanding the underlying mechanisms and more effective strategies for diagnosis, prevention and management are needed.

**Objective:**

We sought to identify the global architecture of blood transcriptomic features of anaphylaxis by integrating expression data from human patients and mouse model of anaphylaxis.

**Methods:**

Bulk RNA-sequencings of peripheral whole blood were performed in: i) 14 emergency department (ED) patients with acute anaphylaxis, predominantly to *Hymenoptera* venom, ii) 11 patients with peanut allergy undergoing double-blind, placebo-controlled food challenge (DBPCFC) to peanut, iii) murine model of IgE-mediated anaphylaxis. Integrative characterisation of differential gene expression, immune cell-type-specific gene expression profiles, and functional and pathway analysis was undertaken.

**Results:**

1023 genes were commonly and significantly dysregulated during anaphylaxis in ED and DBPCFC patients; of those genes, 29 were also dysregulated in the mouse model. Cell-type-specific gene expression profiles showed a rapid downregulation of blood basophil and upregulation of neutrophil signature in ED and DBPCFC patients and the mouse model, but no consistent and/or significant differences were found for other blood cells. Functional and pathway analysis demonstrated that human and mouse blood transcriptomic signatures of anaphylaxis follow trajectories of upregulation of cell movement, migration and neuroinflammatory signalling, and downregulation of lipid activating nuclear receptors signalling.

**Conclusion:**

Our study highlights the matched and extensive blood transcriptomic changes and suggests the involvement of discrete cellular components and upregulation of migration and neuroinflammatory pathways during anaphylaxis.

## Introduction

Anaphylaxis is an acute systemic hypersensitivity reaction that is rapid in onset and usually associated with potentially life-threatening airway, breathing, and/or circulatory compromise, often with skin and mucosal changes (1). It is mostly triggered by food, drugs, or insect stings (1, 2). Anaphylaxis is increasingly a concern at a global level; therefore, further progress in understanding the underlying mechanisms and more effective strategies for diagnosis, prevention and management are needed (1–4).

Although mast cell activation *via* IgE-mediated pathways is postulated to have a pivotal role in anaphylaxis, there has been steady progress in understanding that other pathways, effector cells and mediators potentially contribute to symptoms (1), with evidence for a potential role involving complement, basophils, neutrophils, monocytes/macrophages, platelets, and mediators, such as platelet-activating factor (PAF), cysteinyl leukotrienes (CysLTs), tryptase, and cytokines/chemokines (1, 5–9).

Transcriptomic data relating to anaphylaxis in human subjects, indicate that innate immune pathways (10) and immune cells, such as neutrophils, naïve CD4^+^ T cells, and macrophages are involved in the pathogenesis of human anaphylaxis (11). However, these data are not accomplished or integrated by transcriptomic analysis from mouse models of anaphylaxis, which have been developed to investigate the functions of antibodies, Fc receptors, and effector cells in anaphylaxis (1, 12).

To address this evidence gap, we integrated acute transcriptomic changes (bulk RNA-sequencing) of peripheral whole blood observed during anaphylaxis (i) in patients presenting to the hospital emergency department (ED) with acute anaphylaxis triggered mostly by insect stings, (ii) in peanut-allergic patients undergoing double-blind placebo-controlled food challenge (DBPCFC), and (iii) in a study of iIL9Tg mouse model using the passive antigen-induced anaphylaxis model which mimics food-induced IgE-mediated anaphylaxis (12, 13). Integrated data revealed 1023 genes commonly and significantly dysregulated during anaphylaxis in ED and DBPCFC patients; of those genes, 29 were also dysregulated in the mouse model; and common changes in blood basophils and neutrophils, upregulation of cell movement, migration, and neuroinflammatory signalling, and downregulation of lipid activating nuclear receptors signalling.

## Methods

### Study subjects

#### Emergency department study

We recruited 15 patients (8 female patients; age, 20-79 years) presenting with an acute episode of anaphylaxis to the ED of University Hospital Golnik, Slovenia (6). Reaction severity was graded according to the Mueller criteria (14). We collected blood samples at presentation to the ED and 7-14 days and/or 1 month after the anaphylactic episode (**Table 1**).

**Table 1:** Patients with acute anaphylactic reactions recruited from the hospital emergency department.

No.	Sex	Age	Culprit	Mueller grade	Time from onset of reaction to sampling*	Emergency treatment
1	F	20	European Hornet	4	30 min, 7 d, 1 m	aH1 (2tbl, 2 mg iv) ST (64mg po, 125 mg iv)
2	F	70	Unknown *Hymenoptera*	3	2 h25 min, 14 d, 1 m	aH1 (2 mg iv), ST (500 mg iv)
3	M	71	Yellow Jacket	3	2 h 30 min, 7d, 1 m	aH1 (2 mg iv), ST (125 mg iv)
4	M	57	European Hornet	4	2 h 45 min, 13 d	aH1 (2 mg iv), ST (40 mg iv)
5	F	33	European Hornet	1	4 h, 7 d, 1 m	No drugs administered
6	M	50	Yellow Jacket	4	1 h 20 min, 7 d, 1m	Epi (0.3 mg im), aH1 (2tbl, 2 mg iv), ST (64 mg po, 125 mg iv), bronchodilator (fenoterol 0,5mg, ipratropium bromide 0,2mg)
7	M	48	Honeybee	4	1 h 20 min, 7 d, 1 m	Epi (0.3-0.5 mg im), aH1 (2 mg iv), ST (>40 mg iv)
8	F	55	Honeybee	3	3h 10 min, 7 d, 1 m	Epi (0.3 mg iv), aH1 (2tbl, 2 mg iv), ST (64 mg po, 500 mg iv)
9	M	47	Wasp	3	2 h, 7 d, 1 m	aH1 (2 mg iv), ST (80 mg iv)
10	M	62	European Hornet	4	55 min, 8 d, 1 m	Epi (0.5 mg im), aH1 (2 mg iv), ST (125 mg iv)
11	F	56	Idiopathic	4	<1 h, 7 d, 1 m	Epi (0.3-0.5 mg), aH1 (2mg iv), ST (125 mg iv)
12	F	56	Wasp	4	2 h, 7 d, 1 m	Epi, aH1, ST
13	M	79	European Hornet	3	1 h, 7 d, 1 m	aH1 (2 mg iv), ST (80 mg iv)
14	F	66	Iv analgesic	4	20 min, 7 d, 1 m	Epi (0.3 mg im), aH1 (2 mg iv), ST (80 mg iv)
15	F	56	Honeybee	4	55 min, 7 d, 1 m	Epi (2x 0.5 mg im), aH1 (2 mg iv), ST (125 mg iv)

aH1, H_1_-antihistamine (clemastine); Epi, epinephrine; ST, steroid (methylprednisolone); min, minutes; d, days; m, months. European hornet (Vespa crabro); wasp (Vespula vulgaris; Polistes); honey bee (Apis mellifera).

#### Peanut allergy study (DBPCFC study)

We recruited 11 patients with peanut allergy in whom allergy was confirmed by DBPCFCs as described elsewhere (15). Blood samples were collected prior to the challenge, at the onset of objective symptoms, and 2-4 hours after the challenge (**Table 2**).

**Table 2:** Patients with peanut allergy undergoing double-blind placebo-controlled food challenge to peanut.

No	Sex	Age	Cumulative reaction dose(mg PN protein)	Muellergrade	Sampling time†	Emergency treatment
1	M	31	1333	2	PreFC, at reaction, 2h post	Epi at reaction aH1 and HC post reaction
2	M	15	143	3	Pre FC, at reaction	Epi post sampling
3	F	13	4443	3	PreFC, at reaction, 4h post	Epi, aH1, β2-agonist post reaction
4	M	15	1443	3	PreFC, at reaction, 4h post	aH1 at reaction Epi post reaction
5	F	13	1443	2	PreFC, at reaction, at anaphylaxis, 4h post	Epi at 1h post (time of anaphylaxis), aH1 after sampling at 1h
6	M	9	13	3	PreFC, at reaction, 4h post	aH1, Epi after sampling at time of reaction
7	M	9	143	3	PreFC, at reaction, 40min post	Epi, β2-agonist post reaction
8	M	14	443	3	PreFC, at reaction, 4h post	Epi, aH1, β2-agonist, iv fluids post reaction
9	M	22	133	2	PreFC, at reaction, 2h post	aH1, HC post reaction
10	M	27	63	2	PreFC, at reaction, 2h post	aH1, HC post reaction
11	F	13	143	3	Pre FC, at reaction	aH, Epi, HC, β2-agonist after sampling

aH1, H1-antihistamine; Epi, epinephrine; HC, hydrocortisone; iv, intravenous; PN, peanut. †Whole blood samples were collected into PAXgene blood tubes from a venous cannula sited prior to challenge (FC), at time of reaction, and then at the stated timepoint afterwards.

#### Mouse model of anaphylaxis (Mouse study)

For passive oral antigen-induced anaphylaxis, iIL9Tg mice were sensitised and challenged as previously described (12). In iIL9Tg mice, murine IL-9 is under the control of the intestine-specific promoter of the rat fatty acid–binding protein (iFABPp) gene. This promoter has been extensively used to direct the expression of genes, specifically in enterocytes of the small intestine (16). A consequence of constitutive expression of IL-9 in the SI is a significant increase in mast cell levels predominantly localised to intraepithelial, intercryptic, and lamina propria regions of the SI. We have previously demonstrated that administration of antigen-specific IgE (anti-TNP-IgE) and subsequently challenged with a single oral dose of antigen [TNP-ovalbumin (OVA)] induces an acute allergic reaction characterised by gastrointestinal (GI) and systemic symptoms (17). Briefly, mice were injected intravenously (i.v.) with 10 μg of anti-TNP-IgE (200 μL of saline) with or without IL-4C (recombinant, IL-4–neutralising, anti–IL-4 monoclonal antibody [mAb] complex, 1:5 weight) (anti–IL-4 mAb, clone BVD4-1D11.2 obtained from Fred Finkelman, CCHMC) (17). Twenty-four hours later, mice were held in the supine position and orally gavaged with 250 μL of TNP-Ovalbumin (Ova; 50 mg) in saline (17). Before the intragastric (i.g.) challenge, mice were deprived of food for 3-4 hours. Challenges were performed with i.g. feeding needles (01-290-2B; Fisher Scientific Co., Pittsburgh, PA). For passive anaphylaxis, iIL9Tg mice were injected i.v. with 10 μg of anti-IgE [EM-95; obtained from Fred Finkelman, CCHMC (18)] in 100 μl saline and anaphylaxis assessment performed (12). Hypothermia (significant loss of body temperature) was used as an indication of anaphylaxis. Rectal temperature was measured with a rectal probe and a digital thermocouple thermometer (Model BAT-12; Physitemp Instruments, Clifton, NJ, USA). Temperature was taken prior to the challenge and then every 15 min for 30 or 60 minutes to identify maximum temperature change as previously described (12). Control mice were defined as mice that received anti-TNP IgE and subsequently received saline *via* o.g. and did not experience shock (hypothermia) response 30 minutes following challenge (FC). Mice that experienced a mild to moderate reaction received anti-TNP IgE and subsequently received o.g. OVA-TNP and experienced a 1.5 - 1.9˚C temperature loss within 30 minutes of oral FC. Mice that experienced a severe reaction received anti-TNP IgE and IL-4C and o.g. OVA-TNP and experienced a ≥ 3.7°C temperature loss within 30 minutes of the FC. Peripheral whole blood was drawn from iIL9Tg mice 2 hours following the intragastric challenge.

### Study approval

#### Human studies

This study was conducted in accordance with the amended Declaration of Helsinki. Ethical approval was obtained from the Slovenian National Medical Ethics Committee (ED study) and the London Central Research Ethics Committee (DBPCFC study). All subjects provided written informed consent.

#### Animal studies

All mice were maintained and bred in a barrier facility, and animals were handled under approved Institutional Animal Care and Use Committee protocols at the University of Michigan animal facility.

### RNA sequencing

#### Processing of blood samples and RNA isolation

Whole blood samples were collected into Tempus™ Blood RNA tubes (Thermo Fisher Scientific, Waltham, MA, USA) or PAXgene blood tubes (Qiagen, Hilden, Germany) containing RNA stabilising reagent. Total RNA was isolated using QuickGene blood cell kit (RB-S) and Fujifilm QuickGene-810 automated system (Fujifilm Life Sciences, Tokyo, Japan) or PAXgene Blood miRNA Kit and fully automated QIAcube system (Qiagen), according to the manufacturer’s isolation protocol. The purity of isolated RNA was assessed using the NanoDrop 2000c spectrophotometer (Thermo Fisher Scientific), and integrity was determined on Agilent 2100 Bioanalyzer using RNA 6000 Nano LabChip kit (Agilent Technologies, Santa Clara, CA, USA). All samples used for library preparation had RNA integrity values greater than 7.0. Extracted RNA samples were stored at -80°C before further processing.

#### Sample library preparation

We performed library preparation using the TrueSeq stranded total RNA sample preparation kit (Illumina, San Diego, CA, USA), according to the manufacturer’s protocol. After Ribo-Zero rRNA depletion, the remaining RNA was purified, fragmented, and primed for first strand cDNA synthesis with reverse transcriptase (SuperScript II) and random primers, followed by second strand cDNA synthesis performed in the presence of dUTP. Blunt-ended double-strand DNA was 3’ adenylated, and multiple indexing adapters (T-tailed) were ligated to the ends of the ds cDNA. Fragments with adapter molecules on both ends were selectively enriched with 15 cycles of PCR reaction. 44 libraries were normalised to the final concentration of 10 nM and then pooled in equimolar concentrations. Sequencing was performed on the Illumina Hiseq 2000 sequencing system in 2×100 sequencing cycles using pair-end sequencing mode.

#### RNAseq data analyses

After initial read quality filtering and demultiplexing using Bcl2Fastq v1.8., alignments to the human reference genome (hg19) and mouse genome (mm10) were performed bowtie and tophat alignment softwares. Transcript abundances were then estimated using read counts function in Subread package for R (19), followed by intersample normalisation by variance modelling at the observational level (voom) approach implemented in limma Bioconductor package (20). Differences in gene expression were estimated using linear modelling and significance estimation procedures in limma. Principal component analysis was performed using the built-in function in R 4.0.3. Prior to further calculations, we removed the low-expressed genes by filtering out the genes that displayed counts per million (cpm) values below 1 in over 95% of the samples tested. This enabled removal of genes characterised by the low expression across all the samples, while still retaining potential biologically relevant genes that displayed low expression in a subset of samples, but a higher expression level in others.

The significance estimations were controlled for multiple testing using the false discovery rate approach (FDR) implemented in limma, and genes with FDR values of 0.1 or less were deemed dysregulated (differentially expressed).For comparison of human and mouse transcriptional patterns, we performed the conversion of the gene identifiers using orthology data in the Ensembl database, using biomaRt package for R.

#### Gene set enrichment analyses

In order to gain insights into biological mechanisms during anaphylaxis and to determine whether examined gene sets show statistically significant differences between stages, we performed Gene Set Enrichment Analysis (GSEA). Normalised sample expression levels (normalised RPKM values) of genes were used in the analysis. Gene sets collections (hallmark - H, curated gene sets – C2, ontology gene sets - C5, immunologic signature gene sets - C7) were obtained from the Molecular Signature Database (MSigDB) complied by Broad Institute: http://www.broadinstitute.org/gsea/index.jsp. To assess the statistical significance of the enrichment score, we used 1000 permutations.

#### Immune cell-type-specific gene expression profiles

We generated the gene signatures of immune cells based on the data from published immune cell expression profiling studies. Briefly, we analysed the publicly available immune cell transcriptome data for humans (GSE3982) (21) and mice (GSE37448) (22). For each immune cell type characterised in these studies, we selected genes that performed best at differentiating a specific immune cell type from others characterised in the study (23, 24). For each cell type investigated in the published immune cell profiling datasets, we performed a moderated t-test comparison (limma) of gene expression of the selected cell type and compared it to expression profiles of other cell types analysed in the study. We performed this iteratively across all the cell types investigated in the published datasets. Using this approach, we identified genes that were specifically over-expressed and were therefore considered as characteristic of a specific immune cell type versus other cell types. Only genes that attained a highly significant upregulation were included in each signature (P-value < 10^-12^). GSEA analysis was then used to detect the enrichment of gene sets belonging to each immune signature set. The list of genes included in human and mouse immune cell signatures are provided in the **Supplementary Materials** (**Supplementary Tables 11**, **12**).

#### Functional and pathway analysis

The functional analyses were generated through the use of QIAGEN’s Ingenuity^®^ Pathway Analysis (IPA^®^, QIAGEN Redwood City, www.qiagen.com/ingenuity). DEGs with corresponding expression values were uploaded into the IPA software (Qiagen). The core analysis function included in the software was used to interpret the DEG data, which included diseases and functions, canonical pathways, and upstream transcriptional regulators.

#### Data availability

Raw sequencing and read-count level data were submitted to GEO repository (GSE215184).

## Results

### Study participants

#### Emergency department study

**Table 1** shows detailed information on demographic and clinical characteristics, emergency treatment, and sampling data of 15 ED patients. The reaction was caused by a *Hymenoptera* insect sting in 13 (87%) patients. One patient had a Mueller grade I, 5 a Mueller grade III, and 9 a Mueller grade IV reaction. The median time from the onset of symptoms to peripheral blood sample collection was 1 hour 20 minutes (range 20 minutes to 4 hours). Convalescent samples were collected from all patients median 7 days (range 7 to 14 days) and 14 patients one month after the anaphylactic episode.

#### Peanut allergy study (Peanut-DBPCFC study)

In 11 peanut-allergic individuals, peripheral blood samples were collected prior to DBPCFC. Four patients had a Mueller grade II and 7 a Mueller grade III reaction. According to Mueller grading, the reaction severity was significantly lower in the DBPCFC group compared to the ED group (Median 3 vs 4; P = 0.002; Mann-Whitney U test). Following the objective reaction, further blood samples were obtained from all patients, and in 9, 2-4 hours after reaction. One patient (patient 5) experienced anaphylaxis 1 hour after an initial non-anaphylaxis reaction and therefore had 2 post-reaction samples collected (**Table 2**).

#### Mouse model of anaphylaxis (Mouse study)

Individual transcriptomic analysis was ascertained from the blood of 8 mice with severe IL-4C amplified anaphylaxis within 30 minutes of oral challenge, 8 with mild to moderate anaphylaxis within 30 minutes of the challenge, and 7 control mice, that did not experience shock (hypothermia) response 30 minutes following challenge for baseline comparison (17).

### Anaphylaxis in humans and mice is associated with extensive and common gene dysregulation

1023 genes were commonly and significantly dysregulated during anaphylaxis in ED and DBPCFC patients; of those genes, 29 were also dysregulated in the mouse model.

**Figure 1:**
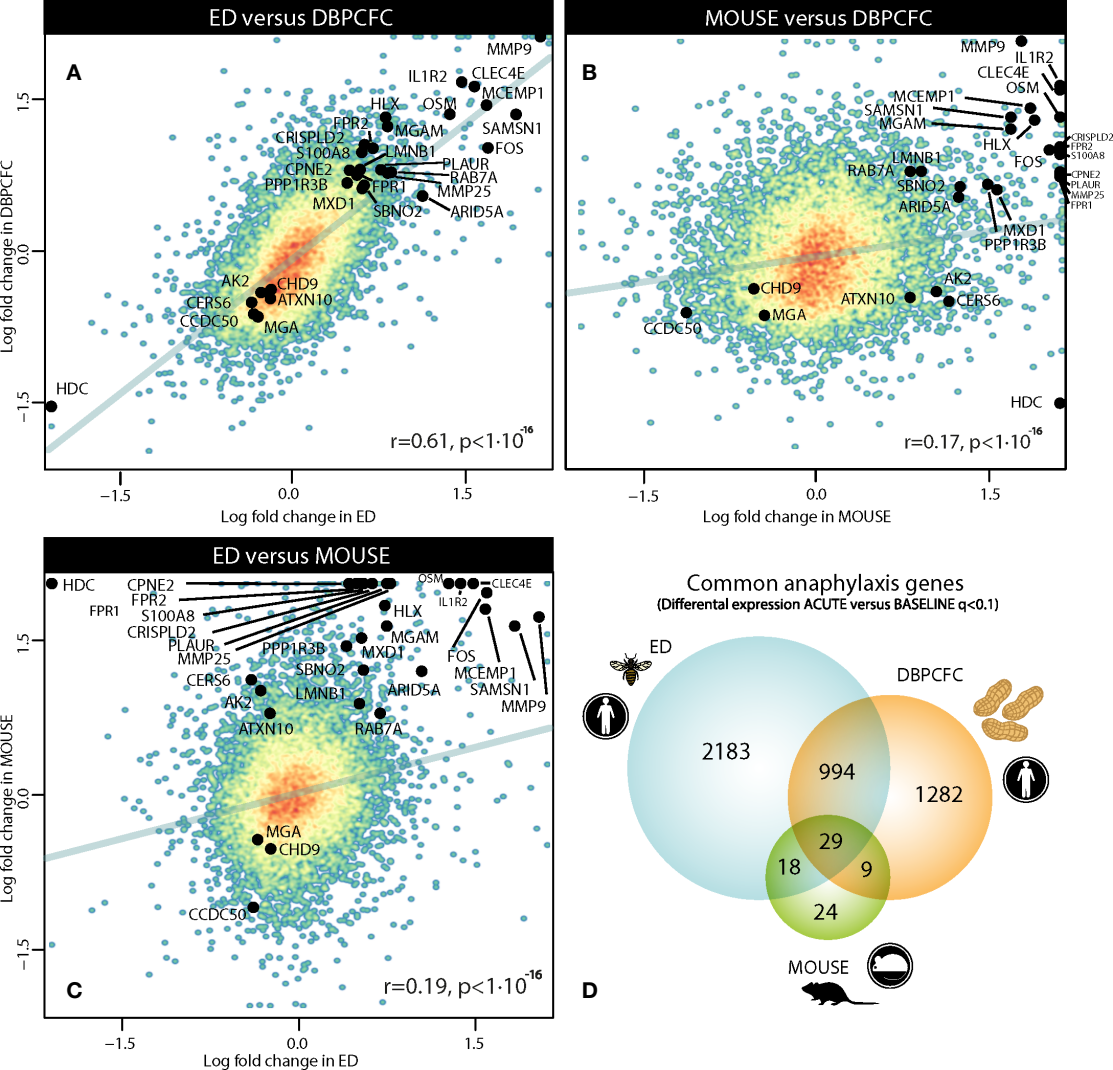
Comparison of gene dysregulation during the anaphylactic episode between human and mouse model of anaphylaxis: **(A)** between ED and DBPCFC study, **(B)** between MOUSE and DBPCFC study, and **(C)** between ED and MOUSE study. Overall 3224, 2314 and 80 genes were differentially expressed in ED, DBPCFC and mouse study group, respectively with **(D)** the overlap of 1023 DEGs for both human situations of anaphylaxis and 29 DEGs for all three study groups. Genes (n=29) differentially expressed in human and mouse model during anaphylaxis are highlighted. ACUTE, acute anaphylactic episode; ED, Emergency Department; DBPCFC, double-blind placebo-controlled food challenge.

We compared gene expression signatures during the anaphylaxis episode between different provoking agents (insect stings and peanut) and human (ED, Peanut-DBPCFC studies) or murine model of anaphylaxis. Therefore, we first compared differentially expressed genes (DEGs) between all three study groups, and found a high correlation in transcriptional response during human anaphylaxis, between ED and DBPCFC (r = 0.61, P < 10^-16^; **Figure 1A**). While only limited correlation was found between human and mouse model (MOUSE and ED (r = 0.19, P < 10^-16^); MOUSE and DBPCFC (r = 0.17, P < 10^-16^; **Figures 1B, C**). Detailed analysis revealed that in the emergency department (ED) study, no differences in RNA expression when comparing the convalescent samples 7 days and 1 month after the acute allergic reaction was detected. On the other hand, significant differences in RNA expression profiles were observed when comparing samples after acute allergic reaction with either 7 days and 1 month after the episode. Therefore, in all downstream analyses, RNA expression after the acute allergic reaction was compared with merged convalescent samples after 7 days and 1 month after the episode, as 3224 differentially expressed gene (DEGs) were found during anaphylaxis in comparison to convalescent state.

When analysing the transcriptional changes in the double-blind placebo-controlled food challenge (DBPCFC) study group, only modest changes in gene expression were detected at the cessation of the challenge in comparison to before, whereas most profound changes were detected when comparing after the challenge with before DBPCFC, where 2314 DEGs were detected. Therefore, for all subsequent analyses, RNA expression after the challenge was compared with the status before the challenge.

In the Mouse study group, we compared RNA expression levels in samples with anaphylaxis (mild and severe) in comparison to mouse samples without anaphylaxis and found 80 DEGs between the two groups. Only modest changes in RNA expression levels were observed between mice with mild and severe anaphylaxis.

To identify the key genes dysregulated during the anaphylactic episode we then analysed which genes are differentially expressed in all three study groups; 1023 genes were commonly and significantly dysregulated during anaphylaxis in ED and DBPCFC patients; of those genes, 29 were also dysregulated in the mouse model (q < 0.1; **Figure 1D**). Top DEGs found only in human anaphylaxis are presented in **Table 3**, while top overlapping DEGs regulated in anaphylaxis are presented in **Table 4**. Details in **Supplementary Tables 1**–**5**. DEGs in peripheral whole blood might reflect alterations in transcriptional regulation or immune cell type composition, both reflecting the pathophysiological mechanism of anaphylaxis (11). Among the six key upregulated drivers of acute peanut allergic reactions recently identified by Watson et al. (11), five, specifically, *KLHL2*, *PPP1R3D*, *PADI4*, *IL1R2*, and *ECHDC3*, were also found to be upregulated (all q<0.05) in both human ED and DBPCFC study. No dysregulation of the *LTB4R* gene was found in our study.

**Table 3:** Top differentially expressed genes (q < 0.1) in human anaphylaxis (ED and DBPCFC).

GeneID	adj.P.Value_ED	logFC_ED	adj.P.Value_DBPCFC	logFC_DBPCFC
*ARG1*	<0.01	1.54	0.01	2.58
*BMX*	<0.01	1.35	<0.01	2.47
*CCR3*	<0.01	-0.82	0.02	-0.66
*CD177*	<0.01	2.10	0.01	3.04
*CDK5R1*	<0.01	1.36	0.01	1.36
*CLEC4D*	<0.01	1.30	<0.01	2.09
*CLEC4E*	<0.01	1.40	0.02	1.53
*CPA3*	<0.01	-2.07	0.02	-1.65
*CST7*	<0.01	1.30	0.02	0.96
*CYSTM1*	<0.01	0.62	0.02	1.03
*DUSP1*	<0.01	1.46	0.01	0.97
*ECHDC3*	<0.01	1.81	0.01	2.40
*FOS*	<0.01	1.51	0.01	0.96
*GATA2*	<0.01	-1.13	0.04	-1.16
*HDC*	<0.01	-2.23	0.04	-1.47
*HRH4*	<0.01	-1.03	0.01	-0.87
*IL18R1*	<0.01	1.43	0.02	1.56
*IL18RAP*	<0.01	1.37	0.03	1.18
*IL1R2*	<0.01	1.30	0.03	1.57
*IL5RA*	<0.01	-1.19	<0.01	-1.61
*IRS2*	<0.01	1.38	0.02	1.40
*KLHL2*	0.02	0.49	0.03	0.78
*MMP9*	<0.01	1.93	0.01	2.13
*MYCL*	<0.01	-1.33	0.04	-0.70
*ORM1*	0.01	1.27	<0.01	2.46
*OSM*	<0.01	1.20	0.01	1.27
*PADI4*	<0.01	1.31	0.01	1.54
*PER1*	<0.01	2.37	0.04	1.94
*PPP1R3D*	0.01	0.39	0.02	0.61
*PTGDR2*	<0.01	-1.21	<0.01	-1.88
*SAMSN1*	<0.01	1.74	0.04	1.27
*SEMA7A*	<0.01	-0.57	<0.01	-1.12

DBPCFC double-blind, placebo-controlled food challenge; ED; emergency department; FC, fold change. Differentially expressed genes are listed in alphabetical order. Downregulated genes are presented in blue and upregulated in red.

**Table 4:** Differentially expressed genes (q < 0.1) in all three study groups (human (ED and DBPCFC) and mouse study groups).

GeneID	adj.P.Value_ED	logFC_ED	adj.P.Value_DBPCFC	logFC_DBPCFC	adj.P.Value_MOUSE	logFC_MOUSE
*AK2*	<0.01	-0.31	0.04	-0.40	0.10	0.99
*ARID5A*	<0.01	0.98	0.07	0.51	0.10	1.17
*ATXN10*	0.06	-0.24	0.06	-0.45	0.06	0.77
*CCDC50*	0.03	-0.37	0.04	-0.60	0.02	-1.07
*CERS6*	<0.01	-0.39	0.01	-0.49	0.06	1.09
*CHD9*	0.01	-0.23	0.03	-0.37	0.10	-0.51
*CLEC4E*	<0.01	1.40	0.02	1.53	0.06	2.25
*CPNE2*	0.05	0.40	0.04	0.75	0.02	3.35
*CRISPLD2*	0.06	0.52	0.03	0.99	0.06	2.91
*FOS*	<0.01	1.51	0.01	0.96	0.06	1.91
*FPR1*	0.01	0.46	0.02	0.70	0.05	2.52
*FPR2*	<0.01	0.59	0.01	0.95	0.06	2.29
*HDC*	<0.01	-2.23	0.04	-1.47	0.01	2.79
*HLX*	0.02	0.69	0.02	1.24	0.10	1.79
*IL1R2*	<0.01	1.30	0.03	1.57	0.02	3.40
*LMNB1*	0.01	0.48	0.04	0.75	0.03	0.86
*MCEMP1*	<0.01	1.50	0.07	1.36	0.02	1.76
*MGA*	0.01	-0.34	0.01	-0.63	0.07	-0.43
*MGAM*	0.02	0.70	0.02	1.16	0.10	1.60
*MMP25*	0.01	0.70	0.08	0.72	0.09	2.16
*MMP9*	<0.01	1.93	0.01	2.13	0.07	1.68
*MXD1*	<0.01	0.50	0.04	0.58	0.02	1.48
*OSM*	<0.01	1.20	0.01	1.27	0.01	3.02
*PLAUR*	<0.01	0.73	0.02	0.73	0.06	2.69
*PPP1R3B*	0.03	0.38	0.04	0.63	0.10	1.41
*RAB7A*	<0.01	0.65	0.05	0.75	0.10	0.77
*S100A8*	0.04	0.50	0.08	0.92	0.08	2.06
*SAMSN1*	<0.01	1.74	0.04	1.27	0.06	1.60
*SBNO2*	0.01	0.52	0.06	0.61	0.09	1.18

DBPCFC double-blind, placebo-controlled food challenge; ED; emergency department; FC, fold change. Differentially expressed genes are listed in alphabetical order. Downregulated genes are presented in blue and upregulated in red.

#### Rapid and common downregulation of basophil and upregulation of neutrophil gene signature in human and murine anaphylaxis

**Figure 2:**
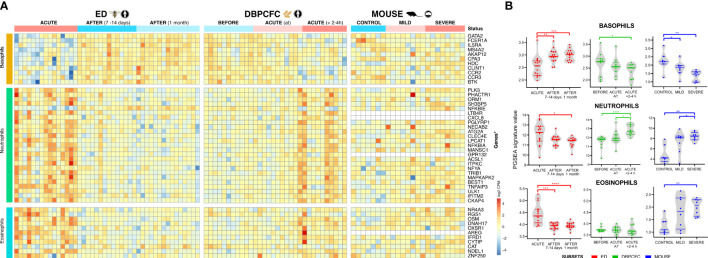
Immune cell-specific profile changes in peripheral blood during anaphylaxis. **(A)** Cell-specific gene signatures of basophils, neutrophils and eosinophils are differentially expressed during anaphylaxis. **(B)** Changes in cell profile in peripheral blood in different time points. No consistent differences were observed in expression signatures of B cells, dendritic cells, macrophages, monocytes, NK cells, T cells (CD4+ and CD8+, Th1 and Th2) (Details in **Supplementary Tables 6, 7**). ACUTE, acute anaphylactic episode; ED, Emergency Department; DBPCFC, double-blind placebo-controlled food challenge. *P <0.05, **P <0.01, ***P <0.001, and ***P <0.0001 by the Mann-Whitney test.

Anaphylaxis involves complex interactions, recruitment, and activities of several different immune cell types. To identify key blood immune cell types participating in anaphylaxis comparative analysis with expression signatures of basophils, B cells, dendritic cells, eosinophils, macrophages, monocytes, neutrophils, NK cells, T cells (CD4^+^ and CD8^+^, Th1 and Th2), were obtained from Molecular Signature Database (MSigDB). Immune cell-type-specific gene expression profiles identified major changes in basophil and neutrophil expression signatures for all three studies: ED, DBPCFC and mouse study of anaphylaxis (**Figure 2**). Eosinophil signatures were different only for ED and mouse studies, but not DBPCFC study. No consistent differences were observed in the expression signatures of other immune cells (Details in **Supplementary Tables 6**, **7**).

During anaphylaxis, we observed a significant decrease in basophil gene signature and an increase in neutrophil specific gene signature, and those changes were evident in all three study groups (**Figures 2A, B**). In the DBPCFC study, trends in basophil and neutrophil changes already become evident at the onset of objective anaphylactic symptoms and then achieved significance at post sampling (**Figure 2B**). Eosinophil specific gene signature was increased only in the ED study, but not under the controlled setting of the DBPCFC study, while in the mouse study group it was increased in mice with severe anaphylaxis (**Figure 2**). The changes in gene expression signatures of basophils and neutrophils might represent either a change in the number of these cells (reflecting movement and/or migration of these cells) and/or changes in distinct basophil and neutrophil subsets with specific/different functional consequences.

#### Gene signatures indicate regulation of cell movement and migration as well as chronic inflammation in integrated human and murine anaphylaxis

**Figure 3:**
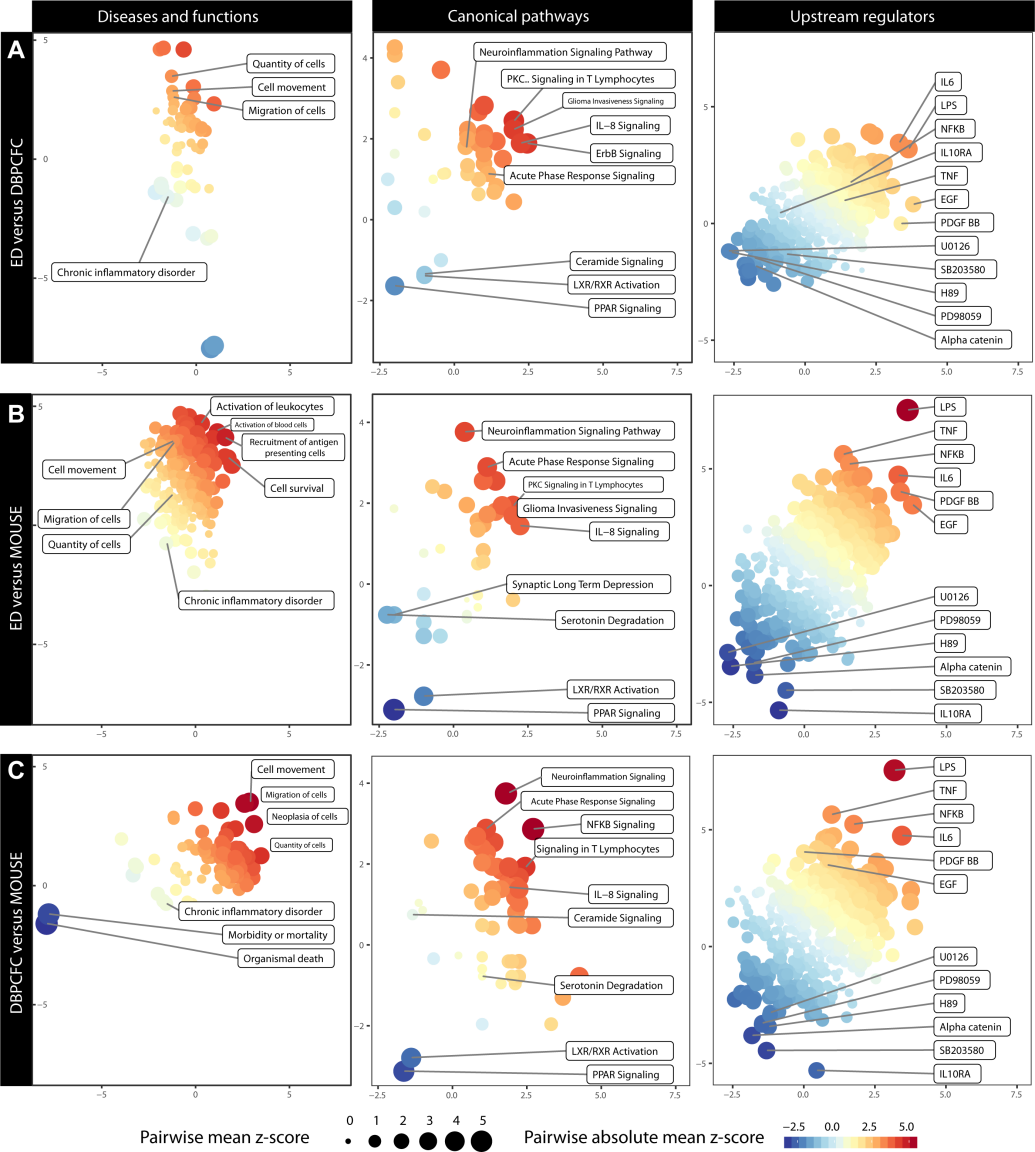
Comparison of altered Diseases and functions, Canonical pathways and Upstream regulators during anaphylaxis between different study groups: **(A)** ED and DBPCFC, **(B)** ED and MOUSE, **(C)** DBPCFC and MOUSE. The plot is based on the z score calculations obtained for a given set of genes using the IPA analysis. The positive values indicate an overall upregulation (red colour) of the genes in a set and negative values indicate a downregulation (blue colour). The colour of the points corresponds to a mean of z scores obtained for the pair of exposures in each panel, and the size of the points corresponds to an absolute average of z scores. The larger and more intensely coloured points, therefore, indicate a more pronounced and analogous dysregulation of a gene set in a given pair of exposures. Details in **Supplementary Table 8** (Diseases and functions), **Supplementary Table 9** (Canonical pathways) and **Supplementary Table 10** (Upstream regulators).

To illustrate interactions, biological context and bio-function among the top DEGs during anaphylaxis, a network and pathway analysis was performed and the top terms enriched in all three study groups and each category are presented in **Figure 3**.

Enrichment of DEGs shows that increased cell movement and migration involving different immune cells represent the most important bio-function in peripheral blood during anaphylaxis. Therefore, as also demonstrated with immune cell-type-specific gene expression profiles, migration and activation of different immune cells – most notably basophils and neutrophils – represent key mechanisms in human and murine anaphylaxis.

Diseases and functions analysis showed that the upregulated DEGs were significantly enriched in biological functions related to cell movement (z score = 2.58, P = 1.19×10^-15^; z score = 2.04, P = 9.58×10^-7^; z score = 2.68, P = 2.64×10^-10^ for ED, DBPCFC and MOUSE study groups, respectively), the quantity of cells and migration of cells (z score = 2.06, P = 9.54×10^-17^; z score = 1.27, P = 1.04×10^-5^ z score = 2.49, P = 6.52×10^-10^ for ED, DBPCFC and MOUSE study groups, respectively), while the downregulated DEGs were enriched in functions related to chronic inflammatory disorders (z score = -2.05, P = 3.84×10^-14^; z score = -0.42, P = 3.57×10^-5^; z score = -0.90, P = 4.31×10^-12^ for ED, DBPCFC and MOUSE study groups, respectively). Details in **Supplementary Table 8**.

#### IPA identifies regulation of acute response, neuroinflammatory and lipid activating nuclear receptors signalling in integrated human and murine anaphylaxis

The most significantly enriched canonical pathways of the upregulated DEGs were acute phase response (*z* scores were 1.98, 2.18, and 1.13, for ED, DBPCFC and MOUSE study groups, respectively), IL-8 signalling (*z* scores were 2.98, 1.21, and 1.34, for ED, DBPCFC and MOUSE study groups, respectively), signalling in T lymphocytes, and neuroinflammatory signalling pathway. For the downregulated DEGs, the most significantly enriched pathways were for lipid-activated nuclear receptors of transcription factors (PPAR (*z* scores were -1.34, -0.78, and -2.24, for ED, DBPCFC and MOUSE study groups, respectively) and LXR/RXR [*z* scores were -0.73, -1.39, and -2.12, for ED, DBPCFC and MOUSE study groups, respectively)] signalling. Further details are in **Supplementary Table 9**.

Upstream regulator analysis revealed that LPS-like response (*z* scores were 3.30, 3.41, and 4.87, for ED, DBPCFC and MOUSE study groups, respectively), TNF (*z* scores were 2.58, 2.16, and 4.01, for ED, DBPCFC and MOUSE study groups, respectively), IL-6, NF-κB (*z* scores were 1.15, 2.40, and 2.00, for ED, DBPCFC and MOUSE study groups, respectively), EGF, and platelet-derived growth factor (PDGF-BB; *z* scores were 3.60, 1.98, and 2.58, for ED, DBPCFC and MOUSE study groups, respectively)) were the most significantly upregulated pathways during anaphylaxis in all three study groups. While alpha-catenin (*z* scores were -2.11, -2.61, and -2.60, for ED, DBPCFC and MOUSE study groups, respectively) and inhibitors of mitogen-activated protein kinases (OU126, PD98059, H89, SB2003580) being among the most downregulated upstream regulators during anaphylaxis. Further details are in **Supplementary Table 10**.

## Discussion

Herein, we have integrated analysis of ED insect sting and controlled DBPCFC peanut human studies with mouse study of anaphylaxis to identify the global architecture of blood transcriptome features of anaphylaxis. The iIL9Tg mouse model permits the induction of an IgE-mediated food allergic reaction following a single oral dose of antigen, as in human anaphylaxis (12, 13). Our study highlights the extensive blood transcriptomic changes, which included dysregulation of 1023 genes in human anaphylaxis and 29 genes that were dysregulated in both human and mouse anaphylaxis, the involvement of blood basophils and neutrophils, and upregulation of cell movement, migration, neuroinflammatory signalling, and downregulation of lipid activating nuclear receptors signalling during anaphylaxis. Notably, the extent and correlations of the transcriptomic changes in human anaphylaxis (1023 genes; correlation coefficient r = 0.61) versus that induced in both human and mouse anaphylaxis (29 genes; correlation coefficients r = 0.17 and 0.19) differ significantly.Among the six key drivers of acute peanut allergic reactions previously identified by Watson et al. (11), five of six, specifically *PADI4*, *IL1R2*, *PPP1R3D*, *KLHL2*, and *ECHDC3*, but not *LTB4R*, were found to be dysregulated in ED and DBPCFC studies, supporting the importance of those genes in human anaphylaxis and the relevance of previous results (11). However, of those 6 genes, only *IL1R2* was dysregulated in the mouse study. Importantly, *IL1R2* was recently identified as one of the genes associated with reaction severity in peanut-allergic children (25). IL-1R2 is expressed by neutrophils, B cells, monocytes, and macrophages, and it is a decoy receptor that neutralises IL-1β, limiting the activation of the pro-inflammatory IL-1 pathway, and influencing the production of different interleukins and NF-κB signalling (11, 26). OSM, oncostatin M, and IL-6 family cytokines are potent neutrophil recruiters (27). OSM stimulates endothelial cell proliferation and migration (28) and is implicated in endothelial signalling (29) and activation (30). Besides, OSM upregulates matrix metalloproteinase-9 (MMP9) through the MAPK/ERK pathway. *MMP9* is an important pro-inflammatory protein, which is involved in activation of mast cells (31) and tissue remodelling (10) and was also identified in our study. The most important source of MMP9 is reported to be activated neutrophils (10, 31). *CCDC50*, and *SBNO2* are involved in NF-κB signalling, *ARID5A, FOS, MGA, MXD1*, and *S100A8* in cellular proliferation, differentiation and apoptosis, and *CD177*, *CPNE2, CST7, FPR1, FPR2, HLX, HRH4, IL5RA, MCEMP1, ORM1, PTGDR2*, and *SBNO2* in immune system regulation and signalling (32).

Multiple lines of evidence suggest that, besides mast cells viewed as key players, discrete cells may contribute to the pathology of anaphylaxis (1, 6, 10, 11, 33). By employing immune cell-type-specific gene expression profiles analysis (23, 24), we showed a rapid change in basophils and neutrophil signatures during anaphylaxis. However, the importance of basophils and neutrophils in the pathophysiology of anaphylaxis is currently unsettled (1). Nevertheless, a rapid and significant decrease in basophil gene signature during anaphylaxis supports speculations that basophil migrate from the circulation rapidly upon allergen challenge (6, 34), possibly to sites of inflammation (7, 35, 36) where they might have an effector role (34). Alternatively this migration might have a protection role by limiting basophil activation in circulation (34). Basophil migration might be mediated by the chemokine CCL2, levels of which are increased during anaphylaxis (6, 7) and which can induce selective migration of human basophils (7, 37).

Recent data suggests that neutrophils are not only important in murine anaphylaxis (1, 8, 38) but might be also important in human anaphylaxis (1, 8). This is supported by our findings of a rapid increase in neutrophil gene signature in both human ED insect sting and controlled DBPCFC peanut studies and consistent with a previous study in peanut-allergic children in which allergen challenge (but not placebo) upregulates neutrophil gene signatures (11). Human and murine neutrophils express several activating FcµRs and human neutrophils also FcϵRI, can release histamine (although in much smaller amounts than mast cells), PAF (which can cause bronchoconstriction, increased vascular permeability, chemotaxis, and degranulation of eosinophils and neutrophils) and CysLTs after stimulation with immune complexes (1, 8). In murine models of anaphylaxis, neutrophil depletion can ameliorate anaphylaxis. A potential contribution of neutrophils during anaphylaxis in humans is supported by increased serum levels of PAF (9) and myeloperoxidase (MPO), the major enzyme stored in neutrophils (39). Recently, increased neutrophil activation markers were also demonstrated during drug, food, and insect-induced anaphylaxis (15, 40).

Aside from basophils and neutrophils, we also observed increased eosinophil gene signatures, but only in patients of the ED study and mice with severe anaphylaxis. Eosinophils express FcϵRI as well as several other receptors (including IL-5 receptor and CCR5) on their surface; their engagement induces eosinophil activation and the release of several mediators including cationic proteins (eosinophil peroxidase, major basic protein, eosinophil cationic protein, eosinophil-derived neurotoxin), lipid mediators (LTC4, PAF), and a variety of cytokines (IL-1, IL-3, IL-4, IL-5, IL-13, GM-CSF, TGF-α/β, TNF-α, chemokines (CCL3, CCL5, CCL11), and neuromodulators (Substance P, vasoactive intestinal peptide) (1, 41–43) – which could sustain or augment the immune response. However, no previous data did show that eosinophils may play a role in anaphylaxis.

Integrated data-driven functional and pathway analysis using IPA disease and function, canonic pathways, and upstream regulator analysis identified cell movement, migration, and neuroinflammatory and lipid activating nuclear receptors signalling as the major blood-related pathways of anaphylaxis. Upregulation of cell movement and migration is consistent with the rapid change in basophils and neutrophil signatures during anaphylaxis and emphasises the potential importance of cellular interactions in anaphylaxis (1, 6, 10, 11, 33). Neurogenic inflammation may be a key driver of some allergic symptoms (44), including rhinitis, conjunctivitis, coughing, bronchoconstriction, airway mucus secretion, dysphagia, altered gastrointestinal motility, and urticaria/angioedema (44). Allergen-induced activation of mast cells could lead to alterations in the function of afferent neurons like intrapulmonary airway C-fiber (45), neurons within the CNS (46, 47), and somatosensory itch fiber in the skin (48), sensory nerve excitability (49) and neuroplasticity (50), increases in synaptic efficacy in autonomic ganglia (51, 52) and stimulation of enteric neurons (53, 54) – an effect which might be more prominent in atopic individuals compared to non-atopic controls (44). Of note, a recent neuronal receptor-based controlling mechanism of anaphylaxis involving the glutamatergic receptor mGluR7was described (55). These data would therefore be consistent with our hypothesis that upregulation of neuroinflammatory signalling pathways might impact neuromodulation in anaphylaxis.

Lipid-activating nuclear receptors signalling, specifically lipid-activated nuclear receptors of transcription factors PPAR and LXR/RXR, was found to be highly downregulated. Ligand-activated transcription factors play important roles in cellular processes such as differentiation, proliferation, survival, apoptosis, and motility in a variety of biological contexts, including inflammation and immune responses (56). LXR and PPAR can antagonise inflammatory pathways through transrepression (57). PPARs can modulate the intensity, duration, and outcomes of inflammatory responses through anti-inflammatory effects (58). PPAR activation also reduces the production of multiple pro-inflammatory mediators, including TNF-α, IL-1α, IL-6, and IL-8. PPAR activation in endothelial cells inhibits endothelial inflammation, improves endothelial function, and inhibits cell proliferation and migration (59). Downregulation/inhibition of these effects thus results in vascular dysregulation. Lipid-activated nuclear receptors also shape macrophage and dendritic cell function (60), and studies in mice have shown that depletion of monocytes/macrophages can reduce anaphylaxis (1).

There was also upregulation of NFκB signalling and lipopolysaccharide (LPS) pathway, upregulation of inflammatory cytokines IL-6, IL-8, TNF-α, and IL-1β, and angiogenic platelet-derived growth factor (PDGF). These cytokines induce the inactivation of cadherin, which mediates cell adhesion and lead to vascular leakage by increased capillary permeability and target several immune cells with the FcγR receptor (involved in acute inflammatory responses) (61). LPS modulates immune responses by interacting with toll-like receptor 4 (TLR4, and can induce both Th1 and Th2 responses (62). PDGF is synthesised and released by platelets upon activation and is increased in children with anaphylaxis; rapid IgE desensitisation, in turn, downregulates PDGF (63).

There are some important limitations to our study. The first and most significant limitation is that we did not perform follow-up functional *in-vitro* experiments and analyses. However, it is important to note that recent studies corroborate possible contributing mechanisms of many of our major discovery genes (*ARG1*, *CPA3*, *FOS*, *GATA2*, *HDC*, *IL18R1*, *IL18RAP*, *MMP9*, *OSM*, and *PER1*) and related them to mast cells or basophils, or other contributing factors. Thus, Do et al. (25) recently identified *ARG1* as a major hub in the network of the genetic and epigenetic architecture of reaction severity in peanut allergy. Dwyer et al. (64) demonstrated that shared mast cell and basophil gene signature networks include ten-fold higher expression of *CPA3* and *GATA2* than other immune cells. Further, the transcription factor GATA2 regulates *HDC* gene expression in mast cells and is required for IgE/mast cell-mediated anaphylaxis (65), and conditional deletions of *HDC* confirm the role of histamine in IgE-mediated anaphylaxis (66). Inhibition of *FOS* expression attenuates IgE−mediated mast cell activation and allergic inflammation (67). Genetic variations at the IL-18 locus (*IL18R1* and *IL18RAP*) were associated with exercise-induced anaphylaxis (68), while *MMP9* and *OSM* provide evidence for the involvement of innate immune pathways (40). OSM is also involved in hyper-IgE syndrome (69). Finally, increased expression of *PER1* inhibits mTORC1 signalling, thus reducing Th1 differentiation and shifting the T-helper subset balance towards Th2 (70). Another important limitation is that due to the relatively small number of included subjects, additional subanalysis by stratifying patients according to reaction severity and trigger could not be performed. Further, bulk RNA-sequencing data was based on a mixed cell population from peripheral blood. Therefore, variations in the immune cell composition of the samples may impact the transcriptional profile. However, to our knowledge, this is the first report of integrated transcriptomic analysis of human anaphylaxis (ED patients with acute reaction, predominantly to *Hymenoptera* venom and patients with peanut allergy undergoing DBPCFC to peanut) and iIL9T mouse model, which mimics food-induced IgE-mediated anaphylaxis. Additionally, it was recently proposed that *Hymenoptera* venom and medications such as quinolone antibiotics can cause non–IgE-mediated anaphylaxis through the activation of Mas-related G protein-coupled receptor X2 (MRGPRX2) on mast cells (71–73). For *Hymenoptera* venom, this novel and intriguing concept is based on a venom constituent mastoparan, which can directly, through MRGPRX2, activate mast cells (74–76). Thus, direct activation of mast cells by mastoparan *via* this receptor (71, 74) may also affect transcriptomic changes observed in our ED patients. However, a broader assessment will be required to replicate these findings (71, 74) and confirm this hypothesis.

In summary, integrative blood transcriptomic analysis in human and murine model of anaphylaxis demonstrated extensive gene dysregulation, mainly involved in NF-κB, MAPK/ERK and endothelial signalling, inflammation, cellular proliferation, differentiation, apoptosis, and immune system regulation and signalling. Importantly, we confirmed, in both human and mouse studies, the rapid changes in blood basophils and neutrophils signatures during anaphylaxis. For the first time, we have identified the upregulation of cell movement, migration, and neuroinflammatory signalling during anaphylaxis. These findings highlight the involvement of distinct effector cells, and complex signalling changes, which reflect cellular movement and interactions, as well as possible implications of the nervous system. Further studies of those pathways might be important for more effective strategies for preventing this disorder and might, in future, also provide novel options for treating anaphylaxis.

## Data availability statement

The data presented in this study are deposited in the GEO repository, accession number GSE215184.

## Ethics statement

This study was conducted in accordance with the amended Declaration of Helsinki. Ethical approval was obtained from the Slovenian National Medical Ethics Committee (ED study) and the London Central Research Ethics Committee (DBPCFC study). All subjects provided written informed consent. Written informed consent to participate in this study was provided by the participants’ legal guardian/next of kin. All mice were maintained and bred in a barrier facility, and animals were handled under approved Institutional Animal Care and Use Committee protocols at the University of Michigan animal facility.

## Author contributions

MR, AM, PK conceived the idea and designed the experiments; MR, AM, PT, KH, MK, AY, SH, AC, BP, PK acquired clinical data, performed experiments, data analysis and interpretation. MR, PK wrote the manuscript. All authors read, revised and approved the final version of the manuscript.
